# Posterior inference of Hi-C contact frequency through sampling

**DOI:** 10.3389/fbinf.2023.1285828

**Published:** 2024-02-22

**Authors:** Yanlin Zhang, Christopher J. F. Cameron, Mathieu Blanchette

**Affiliations:** ^1^ School of Computer Science, McGill University, Montréal, QC, Canada; ^2^ Department of Biochemistry and Goodman Cancer Research Center, McGill University, Montreal, QC, Canada

**Keywords:** Hi-C, posterior distribution inference, Markov random field, Markov chain Monte Carlo, Bayesian inference

## Abstract

Hi-C is one of the most widely used approaches to study three-dimensional genome conformations. Contacts captured by a Hi-C experiment are represented in a contact frequency matrix. Due to the limited sequencing depth and other factors, Hi-C contact frequency matrices are only approximations of the true interaction frequencies and are further reported without any quantification of uncertainty. Hence, downstream analyses based on Hi-C contact maps (e.g., TAD and loop annotation) are themselves point estimations. Here, we present the Hi-C interaction frequency sampler (HiCSampler) that reliably infers the posterior distribution of the interaction frequency for a given Hi-C contact map by exploiting dependencies between neighboring loci. Posterior predictive checks demonstrate that HiCSampler can infer highly predictive chromosomal interaction frequency. Summary statistics calculated by HiCSampler provide a measurement of the uncertainty for Hi-C experiments, and samples inferred by HiCSampler are ready for use by most downstream analysis tools off the shelf and permit uncertainty measurements in these analyses without modifications.

## 1 Introduction

Over the past two decades, scientists have increasingly realized the importance of the three-dimensional (3D) genome structure in cellular activity (Dixon et al., 2012; Rao et al., 2014; Tang et al., 2015; Bonev and Cavalli, 2016; Bonev et al., 2017; Beagan and Phillips-Cremins, 2020). It plays a major role in the enhancer–promoter interaction (Han et al., 2020) and cellular differentiation (Zheng and Xie, 2019). Yet, understanding the 3D genome organization remains in its early stage. Although fluorescence *in situ* hybridization-based high-resolution imaging techniques help us localize selected genomic regions, they are unable to capture chromosome-wide images (Bintu et al., 2018). Until recently, only techniques like Hi-C (Lieberman-Aiden et al., 2009) have permitted the analysis of the whole-genome structure by detecting pairwise genomic fragment interactions. In a Hi-C experiment, DNA fragments in close proximity are ligated and identified through massively parallel sequencing. The number of ligated fragments spanning two genomic regions is stored in a matrix known as a contact map. A Hi-C experiment can only capture a small portion of chromosomal contact pairs. The contact map is, thus, a poor estimation of the chromosomal interaction frequency without a measure of uncertainty. Several probabilistic models have been proposed to combat this uncertainty by modeling Hi-C contact pairs with various distributions (Ay et al., 2014; Varoquaux et al., 2023) in different tasks (i.e., 3D structure inference and significant contact detection).

A key challenge in analyzing Hi-C data is to infer the unbiased interaction frequency matrix, given the Hi-C contact map. There are three widely used approaches: (i) removing bias in the contact map via locus coverage normalization (Rao et al., 2014); (ii) modeling bias in a generalized linear model and finding the bias by solving a Poisson or negative binomial regression (Hu et al., 2012); and (iii) removing bias implicitly via matrix balancing (Rao et al., 2014). We then use the normalized Hi-C contact map in downstream analysis. Researchers usually group consecutive fragments into a fixed-size bin to reduce the sparsity of Hi-C contact maps and perform the analysis at low resolutions. Alternatively, deep learning approaches like HiCPlus (Zhang et al., 2018), HiCNN (Tong and Wang, 2019), and RefHiC-SR (Zhang and Blanchette, 2023) have been proposed to predict a dense high-resolution contact map based on the low-coverage input. Meanwhile, statistical modeling, for instance, HIFI (Cameron et al., 2018), infers the high-resolution contact map by exploiting neighboring information. However, these normalized and enhanced contact maps are point estimations. Many downstream analyses, such as loop and TAD annotation, inherent this uncertainty.

Hi-C is designed to measure whole-genome pairwise interaction frequencies. Hi-C data yield a sparse and noisy observation of the true interaction frequency matrix. Many efforts have been made to better utilize Hi-C contact maps by modeling the observed contact statistically. Generally, the number of observed contacts for a given locus pair is modeled as a random variable that follows a distribution (e.g., Gaussian, Poisson, negative binomial, or zero-inflated negative binomial distribution) with estimated parameters or the hidden state (Rousseau et al., 2011; Hu et al., 2012; Xu et al., 2016a; Xu et al., 2016b; Carty et al., 2017; Carty et al., 2017; Varoquaux et al., 2023). Importantly, neighboring locus pairs are generally assumed to be conditionally independent. In contact map normalization, HiCNorm (Hu et al., 2012), for example, assumes the Hi-C contact follows a Poisson or negative binomial distribution and estimates distribution parameters using a generalized linear model. It aims at removing a proportion of observed contacts that biases could explain. It uses a linear regression to model three primary sources of biases (fragment length, mappability, and GC content) and reports normalized contact as the residual.

Probabilistic modeling of Hi-C contact is also relevant in significant interaction detection. For instance, HiC-DC (Carty et al., 2017) uses a zero-inflated negative binomial log-linear regression to model the zero inflation and overdispersion pattern observed in Hi-C datasets. In addition to approaches that model the interacting pair independently, as neighboring Hi-C contact pairs are highly correlated, HMRFBayesHiC (Xu et al., 2016a) and FastHiC (Xu et al., 2016b) exploit this structural dependence in modeling Hi-C contact maps as a negative binomial distribution. Both models assume that model parameters for each pixel in the Hi-C contact map are determined by its corresponding binary hidden state. The hidden states are defined as an Ising model, with one indicating significant contact. HIFI (Cameron et al., 2018), as a contact map enhancement approach, utilizes a similar structural dependence and uses continuous hidden states to represent true interaction frequencies. In 3D genome inference, BACH (Hu et al., 2013) and HSA (Zou et al., 2016) modify the Poisson regression model described in HiCNorm by adding the spatial distance derived from the predicted 3D structure as a new covariate. MCMC5C (Rousseau et al., 2011) models the contact frequency as a Gaussian distribution with parameters derived from the spatial distance.

Here, we introduce HiCSampler, a Markov random field (MRF) model that provides posterior inference of the Hi-C interaction frequency for Hi-C read count data through Markov chain Monte Carlo (MCMC) sampling (Robert et al., 1999). HiCSampler models the interaction frequencies by taking their structural dependencies into consideration and approximates their posterior distribution by producing a collection of interaction frequency matrix samples.

## 2 Materials and methods

### 2.1 Overview of HiCSampler

We model the observed Hi-C contact counts *o*_*ij*_ for the contact pair (*i*, *j*) as a sample drawn from a Poisson distribution *Pois* (*o*_*ij*_|*λ* = *b*_*ij*_ *t*_*ij*_) conditional on systematic bias *b*_*ij*_, as well as the unobserved random variable *t*_*ij*_, the true relative interaction frequency. *b*, representing the nuisance variation in Hi-C observations due to the sequencing efficiency and mappability, is an observed matrix computed as the outer product of the locus-specific bias vector $\vec{b}$ . $\vec{b}$ captures variation due to one-dimensional features such as the fragment effective length, mappability, and GC content; it is estimated with ICE (Imakaev et al., 2012). The true relative contact frequencies *t* are dominated by structural features such as the genomic distance, topologically associating domains, and loops. We encode these priors on *t* as local potentials *ϕ*(*t*_*ij*_) and pairwise potentials *ϕ*(*t*_*ij*_, *t*_*i*′*j*′_) in a Markov random field T . *ϕ*(*t*_*ij*_) encourages *t*_*ij*_ to come close to the genomic distance-dependent expectation *g*_*ij*_. *g*_*ij*_ is predicted by a generalized linear model fitting with read counts, bias, and genomic distance between contact pairs. *ϕ*(*t*_*ij*_, *t*_*i*′*j*′_) penalizes sharp changes between neighboring contacts and is used to model local structural constraints on contact frequencies. Variances reflecting the uncertainty of distance-based expectation and the smoothness of the true interaction frequency are estimated from the Hi-C data. We approximate the posterior distribution of the latent variable *t*_*ij*_ by conducting adaptive MCMC sampling (Givens and Jennifer, 2012) on $p\left(t_{ij}|b_{ij},o_{ij},T\backslash t_{ij}\right)$ .

### 2.2 Modeling biases

The observed read count deviates from the contact probability due to the existence of biases introduced by sequencing such as the effective fragment length, GC content, and mappability. Bias correction methods producing a normalized contact map (and a bias vector) are routinely used in Hi-C contact map analysis. Among these normalization methods, ICE (Imakaev et al., 2012) produces a normalized interaction frequency matrix *o*_norm_ and bias vector $\vec{b}$ via iterative correction under the assumption of equal visibility of each region. To model the effect of sequencing bias, we incorporate the learned bias $\vec{b}$ into our model directly since the bias and true interaction frequency satisfy *o*_*ij*_ ∼Pois (*b*_*i*_ *b*_*j*_ *t*_*ij*_).

We prefer a two-phase approach where biases are first estimated using existing approaches and then used in the sampling of *t*, rather than a joint sampling approach, because the former enables easy, flexible, and accurate bias estimation from the whole contact map. This also makes HiCSampler easier to include into existing Hi-C data analysis pipelines.

### 2.3 Modeling Hi-C contact using Poisson regression

We first describe a Poisson regression approach to model Hi-C contact frequencies by assuming independence among neighboring pixels in Hi-C contact maps, which is used to define the local potential of our Markov random field. As previously discussed, the Hi-C contact frequency for a given locus pair (*i*, *j*) is mainly affected by the genomic contact distance and various biases. We model the Hi-C contact as *o*_*ij*_ ∼Pois (*b*_*i*_ *b*_*j*_ *g*_*ij*_). *g*_*ij*_ is described with a log-linear model Eq. 1:

$$ln\left(g_{ij}\right)=w_{0}+B\left(\left|i-j\right|\right).$$ (1)

Similar to HiC-DC (Carty et al., 2017), we model the relationship between the normalized contact frequency and genomic distance |*i* − *j*| as a basis spline function *B* with knots defined as 0, 25%, 50%, and 75%, and 100% of the maximum genomic distance in the analysis. This B-spline allows our model to better capture the relationship between genomic distance and interaction frequencies.

Defining *u*_*ij*_ = *b*_*i*_ *b*_*j*_ *g*_*ij*_, we can derive an equivalent model *o*_*ij*_ ∼Pois (*u*_*ij*_), and *u*_*ij*_ is described as Eq. 2

$$ln\left(u_{ij}\right)=w_{0}+B\left(\left|i-j\right|\right)+ln\left(b_{i}b_{j}\right),$$ (2)

where *w*_0_ is the intercept term. We train the model based on 10% of contact pairs randomly sampled from the contact map with a maximum likelihood estimation. The expectation of diagonal-wise normalized true interaction frequency *g*_*ij*_ can be calculated as $g_{ij}=e^{w_{0}+B\left(\left|i-j\right|\right)}$ with the learned parameters by setting *b*_*i*_ *b*_*j*_ = 1.

### 2.4 Markov random field modeling of the true interaction frequency

An MRF is an undirected graph, where each node is associated with a random variable, and edges denote dependencies between random variables. In the context of Hi-C contact modeling, we define two types of random variables for a given contact pair (*i*, *j*): *t*_*ij*_ represents the true interaction frequency and *o*_*ij*_ represents the number of observed contacts. *o*_*ij*_ is conditionally independent of *O*_\*ij*_, given *t*_*ij*_, and *p* (*o*_*ij*_|*t*_*ij*_) ∼ *Poisson* (*λ* = *b*_*i*_ *b*_*j*_ *t*_*ij*_). Similar to HMRFBayesHiC (Xu et al., 2016a) and HIFI (Cameron et al., 2018), we model the interaction frequency as a Markov random field on a second-order neighborhood system. However, the second-order neighborhood approach shows potential to obscure domain boundaries. Following HIFI (Cameron et al., 2018), for a given contact pair (*i*, *j*) and its neighboring contact pair (*i*′, *j*′), if there is a sharp horizontal or vertical transition characteristic of a domain boundary, we remove the contact pair (*i*′, *j*′) from the Markov blanket of the contact pair (*i*, *j*). To identify sharp transitions, we performed a Kolmogorov–Smirnov test to detect significant changes between interaction frequencies residing on one side of a potential boundary to those on the other side. As neighboring contacts are correlated, we define the pairwise potential as Eq. 3

$$\phi \left(t_{ij},t_{i^{\prime }j^{\prime }}\right)=e^{\frac{{\left(log\left(t_{ij}\right)-log\left(t_{i^{\prime }j^{\prime }}\right)\right)}^{2}}{\sigma_{ij}^{2}}}.$$ (3)

In addition, we defined the local potential as Eq. 4

$$\phi \left(t_{ij}\right)=e^{\frac{{\left(log\left(t_{ij}\right)-log\left(g_{ij}\right)\right)}^{2}}{\omega_{ij}^{2}}}.$$ (4)

Hence, the joint distribution of the interaction frequency *t* is Eq. 5

$$p\left(t\right)\propto \underset{i,j}{\prod }\phi \left(t_{ij}\right)\underset{\left(i,j\right)∼\left(i^{\prime },j^{\prime }\right)}{\prod }\phi \left(t_{ij},t_{i^{\prime }j^{\prime }}\right).$$ (5)

We infer the posterior distribution via MCMC sampling according to Eqs 6, 7

$$p\left(t|o\right)\propto p\left(o|t\right)p\left(t\right),$$ (6)

$$=\underset{i,j}{\prod }p\left(o_{ij}|t_{ij}\right)p\left(t\right).$$ (7)

Hyperparameters *σ* ^2^ and *ω* ^2^ encode our beliefs on the strength of dependencies between neighborhoods and with relative genomic distance, which are dataset-dependent, and can be estimated from observations. Given that ICE normalized the Hi-C contact map as a point estimation of the interaction frequency matrix, we estimated $\omega_{ij}^{2}$ as the variance of normalized interaction frequencies with genomic distances equal to |*i* − *j*|. $\sigma_{ij}^{2}$ is estimated as the variance of pairwise differences among normalized interaction frequencies within a 17 × 17 square centered at (*i*, *j*). In our experiment, we observed that at a resolution of 5 kb, HiCSampler demonstrates a comparable performance across a wide range of window sizes (i.e., from 3 × 3 to 21 × 21), with 17 × 17 yielding the best fit on the test set. To analyze Hi-C data at other resolutions, users can split the data into training and test sets and select the window size that yields the highest likelihood on the test set via a grid search over a range of window sizes. In this estimation, $\sigma_{ij}^{2}\neq \sigma_{i^{\prime }j^{\prime }}^{2}$ causes *ϕ*(*t*_*ij*_, *t*_*i*′*j*′_) to differ from *ϕ*(*t*_*i*′*j*′_, *t*_*ij*_). To fix it, we use $\max\left(\sigma_{ij}^{2},\sigma_{i^{\prime }j^{\prime }}^{2}\right)$ as the variance of pairwise potential instead.

In HiCSampler, MRF serves as the prior in our model, allowing us to capture the frequently observed local smoothness and interaction frequency decay in Hi-C contact maps. Furthermore, our empirical Bayes approach empowers HiCSampler to model the non-stationary mean and variance in interaction frequencies across a Hi-C contact map. Recognizing the substantial impact of the prior distribution on the posterior distribution, we conducted experiments exploring alternative priors, including 1) uniform prior; 2) Gaussian prior modeling interaction frequency decay; and 3) a modified MRF with a fixed *σ* ^2^. These investigations enhance our understanding of the sensitivity of the model to different prior specifications.

### 2.5 MCMC sampling

MCMC is a strategy to iteratively draw samples from a given distribution. Compared with other sampling techniques, it only requires the knowledge of an object’s probability up to a constant. MCMC actually consists of a group of algorithms, all of them conducting the sampling by constructing a Markov chain with a unique stationary distribution equivalent as the target distribution. HiCSampler uses the Metropolis–Hastings-within-Gibbs algorithm (Robert et al., 1999), the most popular MCMC method for high-dimensional data sampling.

The original Gibbs sampler involves sampling from the conditional distribution *P* (*t*_*ij*_|*t*_\*ij*_, *o*), which is impractical in our model. Hence, we utilize a one-step Metropolis–Hastings algorithm as a single Gibbs update during sampling (Robert et al., 1999). We denote the proposal distribution as $p\left(t_{ij}^{*}|t_{ij}\right)$ and the Metropolis–Hastings acceptance ratio as $A\left(t_{ij},t_{ij}^{*}\right)=min\{1,\frac{p\left(t_{ij}^{*}|t\backslash t_{ij}^{*},o\right)}{p\left(t_{ij}|t\backslash t_{ij},o\right)}\}$ , and Eq. 8

$$p\left(t_{ij}|t\backslash t_{ij},o\right)\propto \phi \left(t_{ij}\right)Pois\left(o_{ij};t_{ij}\right)\underset{\left(i^{\prime },j^{\prime }\right)∼\left(i,j\right)}{\prod }\phi \left(t_{ij},t_{i^{\prime }j^{\prime }}\right).$$ (8)

We initialize the true interaction frequency matrix *t* with uniformly distributed random numbers in [0,1]. We then sampled from the Markov chain by iterative sampling true interaction frequencies. We start to collect samples after the chain converges to the stationary distribution (mixing), as described below.

#### 2.5.1 Adaptive proposal and auxiliary variables

We set the proposal distribution $p\left(t_{ij}^{*}|t_{ij}\right)$ for each contact pair as a Gaussian distribution centered at the current value. The determination of the Gaussian variance is challenging as both overly narrow and overly wide distributions make the sampler inefficient. We propose an adaptive approach by enabling the sampler to automatically tune the variance of the proposal distribution during the burn-in period. We initialize the variance to 1 for each proposal distribution and then adjust the variance periodically to maintain the acceptance rate around 0.234, which is the optimal acceptance rate under general conditions (Roberts et al., 1997). After the burn-in period, the variance is fixed to prevent the chain from deviating from the target distribution (Robert et al., 1999).

Since a true interaction frequency *t*_*ij*_ is a non-negative random variable, proposing candidates from the Gaussian distribution may result in unnecessary negative proposals. We introduce an auxiliary variable *u*_*ij*_ such that $t_{ij}=e^{u_{ij}}$ to increase the acceptance rate of HiCSampler. In practice, we draw new samples on *u*_*ij*_ space and transform it into *t*_*ij*_ by the one-dimensional change in the variable.

#### 2.5.2 Assessing mixing

The first iterations of MCMC (burn-in) are dependent on the initialization and do not represent proper samples from the target distribution; thus, they are discarded. However, the determination of the length of the burn-in is difficult, and although several approaches exist, none of them provide entirely reliable diagnostics. Following the practice in MCMC5C (Rousseau et al., 2011), we run in parallel two independent and randomly initialized chains. We define the difference between two interaction frequency matrices as the root mean square error (RMSE) for corresponding contact pairs, and track the inter- and intra-chain interaction frequency differences from samples collected every *k* iterations (*k* = 50). We claim that the chains mixed after *K* iterations (*K* ≥ 10*k*) if the mean for inter- and intra-chain RMSEs from the past 10 collected samples is within 10% of each other.

The samples collected from MCMC after the burn-in phase are considered independently distributed. However, consecutive draws are dependent according to the Markov property. This is called auto-correlation in the literature. To alleviate it, we collect samples every *k* iterations. The mean and variance are further calculated at the end of the sampling.

#### 2.5.3 Speed optimization

Within one Metropolis–Hastings step, the evaluation of pairwise potentials contributes most to the computational workload as it requires accessing eight neighboring entries and computing eight different Gaussian functions. To speed up the overall calculation, a natural strategy is to parallelize conditionally independent Metropolis–Hastings steps. Parallel programming has served as a routine in scientific computing for decades, and it is proved to be efficient in many MCMC-based applications. As shown in Eq. 8, to sample *t*_*ij*_ from the posterior distribution, we only need to access random variables inside its Markov blanket, along with the observation *o*_*ij*_. This means we can simultaneously sample conditionally independent interaction frequencies with the described sampling algorithm. For a given *n* × *n* contact map, we developed a shared-memory multi-threaded algorithm, in which each thread conducts element-wise sampling on a *m* × *m* submatrix (*m* = 200 and *m* ≪ *n*).

Although our parallel implementation speeds up the sampling procedure linearly, conducting inference on a whole contact map at a high resolution still encounters a heavy computational burden. To further speed up HiCSampler by eliminating unnecessary computations, we limit the computation to contact pairs that are within a given maximum genomic distance (i.e., 5 Mb). Despite this, the inference remains computationally intensive. For example, it requires 15 GB memory and 5.1 h to produce 500 samples for human chromosome 14 at a resolution of 5 kb using 10 parallel threads on an i7-8700 CPU. Our optimized HiCSampler can analyze Hi-C contact maps at a resolution of 5 kb on a desktop computer; however, applying it to analyze data at a higher resolution, such as micro-C XL data, is still challenging and requires users to perform the analysis on a dedicated server with a large number of CPU threads.

### 2.6 Hi-C dataset

We downloaded the processed Hi-C datasets for the GM12878 cell line generated by Rao et al. (2014) from the GEO data repository (accession number: GSE63525). We extracted *cis-*interacting read pairs and saved them as tab-separated values for analysis. We performed contact map normalization using ICE (Imakaev et al., 2012; Servant et al., 2015).

## 3 Results

### 3.1 Probabilistic modeling and Bayesian inference of Hi-C data

**FIGURE 1:**
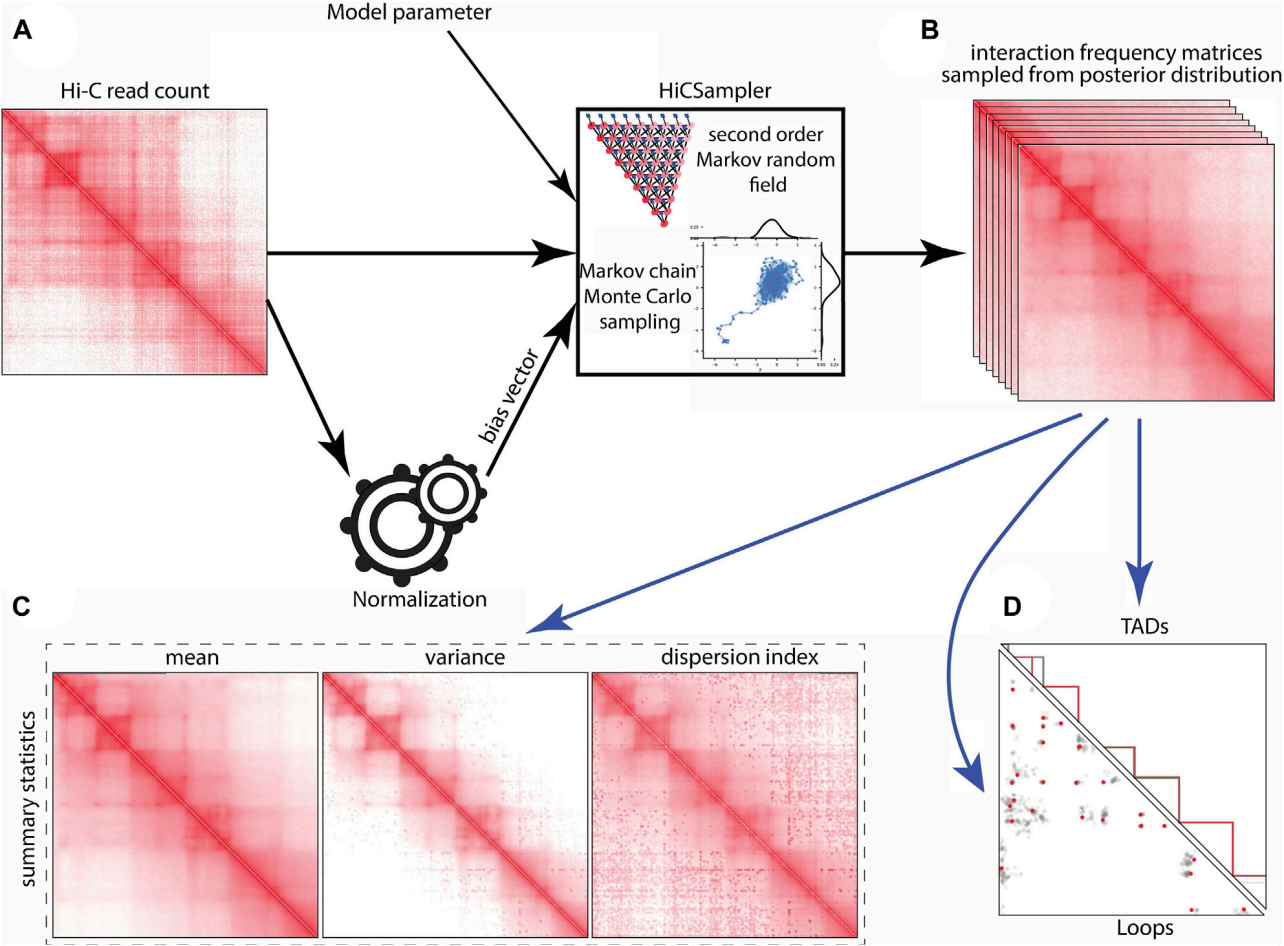
HiCSampler overview. Black arrows indicate essential HiCSampler steps; blue arrow indicates possible downstream analyses based on samples produced by HiCSampler. HiCSampler takes as input **(A)** a Hi-C read count matrix, model parameters, and a bias vector estimated using existing approaches (e.g., ICE). **(B)** It samples from the approximate posterior interaction frequency matrix by Bayesian inference using MCMC. **(C)** Samples inferred with HiCSampler are ready to use for measuring the uncertainty of the interaction frequency with summary statistics (mean, variance, and dispersion index). **(D)** They can also be used to estimate the uncertainty of Hi-C-derived predictions such as TADs and loops.

The outcome of a Hi-C experiment is a read count matrix *o*, whose rows and columns correspond to pre-specified genomic loci, where *o*_*i*,*j*_ is the number of contacts (i.e., read pairs) mapped to the locus pair (*i*, *j*). In this work, we only consider intra-chromosomal contacts and handle each chromosome individually. The matrix *o* depends probabilistically on the unobserved true relative interaction frequency matrix *t*, where *t*_*i*,*j*_ is defined as the proportion of ligation products made of fragments *i* and *j* in the Hi-C library. Note that as the number *n* of read pairs sequenced increases to infinity, and with sequencing biases corrected appropriately, the normalized *o*_*i*,*j*_ converges to *t*_*i*,*j*_. However, since the sequencing coverage is low in practice, the normalized *o*_*i*,*j*_ is a relatively poor estimate of *t*_*i*,*j*_. HiCSampler (Figure 1) aims to infer the posterior distribution of *t* based on observation *o*: Pr [*t*|*o*] ∝ Pr [*o*|*t*] Pr [*t*]. Assuming that a proper prior probability distribution Pr [*t*] and conditional probability distribution Pr [*o*|*t*] are available, this represents the richest possible description of our knowledge of *t*, given *o*. HiCSampler takes the Hi-C contact map, bias vector inferred with ICE normalization (Imakaev et al., 2012; Servant et al., 2015), and model parameters as input and outputs a set of interaction frequency matrices {*s*_1_, *…*, *s*_*n*_} sampled from Pr [*t*|*o*] using the MCMC (Robert et al., 1999) method (Figures 1A, B). Each of the samples is a possible interaction frequency matrix resulting from the Hi-C contact map observed. To measure the uncertainty of interaction frequency in a Hi-C experiment, we summarize sampled interaction frequency matrices as the mean, variance, and dispersion index matrices (Figure 1C). The interaction frequency samples can also be used as input to off-the-shelf Hi-C analysis tools, e.g., to estimate the variability of TAD and loop annotations (Figure 1D).

The MCMC process is time-consuming. Here, we focused on analyzing a randomly picked genomic region enriched with topologically associating domains (chr14:50–60 Mb) to evaluate HiCSampler. We applied HiCSampler to infer 10,000 posterior samples for a Hi-C contact map that contains 300 million read pairs derived from GM12878 cells (Rao et al., 2014).

### 3.2 HiCSampler infers posterior distributions

**FIGURE 2:**
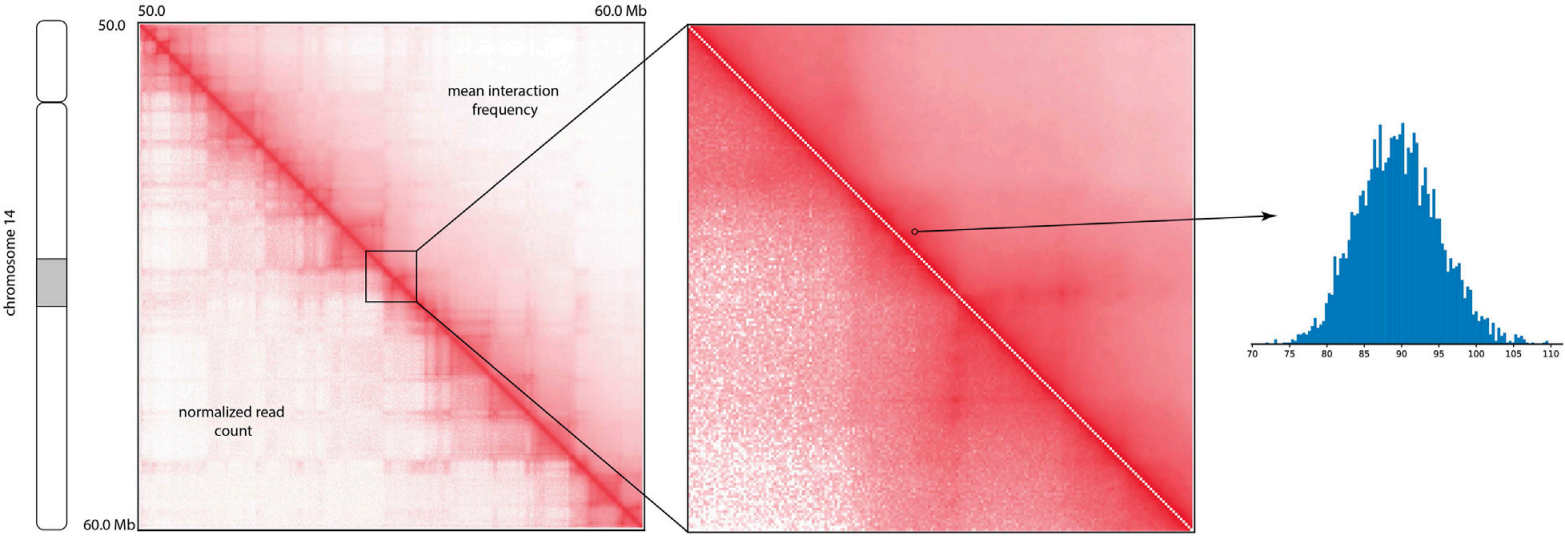
Comparison of the mean of the posterior interaction frequency inferred by HiCSampler and normalized Hi-C read count for a particular region (50–60 Mb) of human chromosome 14 at 5 Kb resolution. The histogram demonstrates posterior distribution for a contact pair approximated using HiCSampler.

**FIGURE 3:**
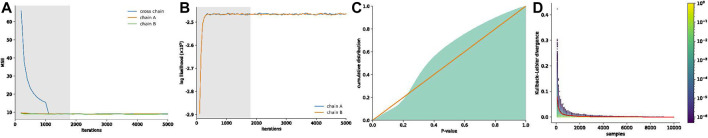
MCMC convergence and posterior predictive checks of HiCSampler. **(A)** Mean square error between within- and across-chain samples. **(B)** Log-likelihood of posterior samples. Burn-in iterations detected by HiCSampler in **(A,B)** are highlighted in gray. **(C)** Cumulative *p*-value distribution for the observed read count matrix, given the inferred posterior distribution. A perfectly uniform distribution would match the red line. **(D)** Distribution of the KL divergence between marginal posterior distributions for each contact pair inferred from different sample sizes and that obtained from the full set of 10,000 samples. The color scale represents the density of the KL-divergence. The red line represents the average KL divergence for all contact pairs.

The visual comparison of the mean of the posterior distribution inferred by HiCSampler and the combined Hi-C contact map (Rao et al., 2014) for the selected region illustrates that HiCSampler can infer highly predictive posterior distribution (Figure 2). Following MCMC5C (Rousseau et al., 2011), we simultaneously run two randomly initialized Markov chains that are compared to determine convergence (see *Methods*). The log-likelihood of posterior samples and the mean square error between within- and across-chain samples for the first 5,000 iterations indicate that HiCSampler can converge to the stationary distribution (Figures 3A, B) of the true interaction frequency within 2,000 burn-in iterations. Additionally, we performed a posterior predictive check by calculating the *p*-values of the observed Hi-C read count conditional on the inferred posterior distribution. We observed that the *p*-values are nearly uniformly distributed, indicating that our model is appropriate (Figure 3C). Using fewer samples to infer the posterior distribution reduces the running time but may lead to inaccurate inference. We then evaluated the accuracy of approximating the posterior distribution using fewer samples. We treated the posterior distribution inferred from 10,000 samples collected after the burn-in iteration as the gold standard and compared distributions approximated with fewer samples to this gold standard. We observed that collecting more samples from HiCSampler improves the accuracy of the posterior inference. As shown in Figure 3D, we can terminate the MCMC process after collecting several hundred samples since the improvement in accuracy is negligible beyond 500 samples. These posterior predictive checks and convergence diagnosis do not guarantee the accuracy of our model but helps us detect potential flaws.

To further investigate the accuracy of HiCSampler, we compared HiCSampler against three other models (see *Methods*) by evaluating the log-likelihood of five different test sets based on the posterior distribution inferred from the training set. We created the training sample and test samples by independently downsampling a Hi-C contact map with 500-M contact pairs to the downsampled data with 250-M contact pairs. Since both HiCSampler and the MRF with the fixed *σ* ^2^ model have hyperparameters that need to be tuned, we used a grid search to set the hyperparameters in both models by evaluating model performance on a test contact map. Following a previous work (Kruschke, 2010), we approximated the log-likelihood of a test sample as the mean log-likelihood of a test sample conditional on 100 posterior samples inferred by HiCSampler. As shown in Table 1, HiCSampler achieved the best log-likelihood on all test samples.

**TABLE 1:** Log-likelihood of individual test data.

Model	Test replicates				
	1	2	3	4	5
HiCSampler	**−1027454**	**−1027658**	**−1027657**	**−1027658**	**−1027739**
Uniform prior	−3574638	−3574707	−3574812	−3574247	−3574339
Gaussian prior	−1283286	−1283381	−1283537	−1283337	−1283330
MRF with fixed *σ* ^2^	−1036005	−1036214	−1036227	−1036189	−1036264

Bold values indicate the best model in each test replicate.

### 3.3 HiCSampler enables quantifying uncertainty in TADs and loop calling

**FIGURE 4:**
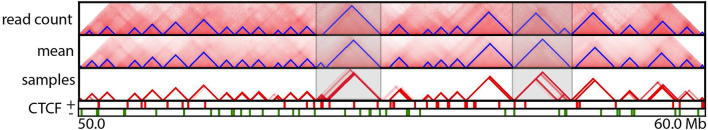
TAD annotations for a particular region (50–60 Mb) of human chromosome 14 at 5 Kb resolution. Panels from top to bottom correspond to TopDom-annotated TADs inferred from the normalized combined read count matrix, mean posterior interaction frequency matrix, and 100 posterior samples; occupancy of forward and reverse CTCF-binding sites. The two highlighted regions exhibit substantial differences among TADs predicted from the normalized Hi-C read count matrix and the mean posterior matrix. This variability was also observed among TADs predicted from posterior samples in these regions.

**FIGURE 5:**
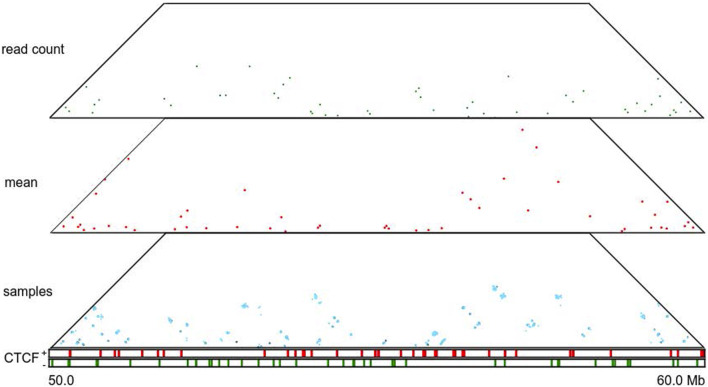
Loop annotations for a particular region (50–60 Mb) of human chromosome 14 at 5 Kb resolution. Panels from top to bottom correspond to HiCCUPS-annotated loops inferred from the combined normalized read count matrix, mean posterior interaction frequency matrix, and 100 posterior samples; occupancy of forward and reverse CTCF-binding sites.

A Hi-C contact map is a point estimation of the true genomic interaction frequency matrix. Thus, it lacks measurements of uncertainty. TADs and loops called based on Hi-C contact maps are also inherently point estimations and likewise come with little quantification of uncertainty. HiCSampler enables studying the variability of called TADs and loops. To illustrate this, we used HiCSampler to sample 100 interaction frequency maps from Pr [*t*|*o*] and annotated TADs and loops from each sample with TopDom (Shin et al., 2016) and HiCCUPS (Durand et al., 2016). Within the 10-Mb region we studied (chr14:50–60 Mb), TopDom identified 24 TADs from both the high-coverage combined normalized read count matrix (Rao et al., 2014) and the mean of the posterior matrix. However, the two sets of annotations are slightly different (Figure 4). These predicted domain boundaries are enriched for CTCF-binding sites. We also identified ∼24 TADs from each of the 100 posterior samples. The 100 sets of TAD annotations largely overlap, and a large portion of these annotations are identical to TADs annotated from the combined normalized read count matrix. Variability in TAD annotations is primarily observed in terms of the exact location of domain boundaries. These observations indicate TAD annotations are generally robust, but the uncertainty in Hi-C contact maps causes an uncertainty in the precise location of TAD boundaries. Among all TAD boundaries annotated from the 100 samples, only 12.5% are detected in all 100 samples; 45% are detected in more than 90% of samples; and 77% are consistently detected from at least half of the samples. We then performed a similar analysis for loop annotations. Compared to TAD annotations, loops annotated from posterior samples have a higher degree of variability. Most of the peaks are consistent in less than 35% of posterior samples (Figure 5). This suggests that loop annotation with HiCCUPS is quite brittle.

## 4 Discussion

Hi-C and its derivatives are widely used to study three-dimensional conformations of chromosomes. Many efforts have been invested in advancing biochemical protocols and data analysis tools, aiming at estimating the interaction frequency accurately and efficiently. Yet, few analytical approaches tackle the measurement uncertainty in interaction frequency estimates or in annotation of loops and TADs. We address this problem by introducing HiCSampler, a Markov random field approach for statistical contact map analysis. HiCSampler is capable of inferring the posterior distribution of the true interaction frequency, conditional on some observed Hi-C read count data. In our experiment, we focus on high-resolution Hi-C data analysis at 5 kb, but HiCSampler can be applied to analyze Hi-C data at different resolutions. The only hyperparameter that might be sensitive to the data resolution is the window size that we used to estimate the variance parameter in the pairwise potential function. As discussed above, this hyperparameter can be determined by evaluating the model on a test set. We believe HiCSampler is an important complement to the existing HiC toolset. It will easily be integrated in existing Hi-C data analysis pipelines, enabling well-grounded estimates of uncertainty of any type of downstream annotation tasks based on off-the-shelf annotation tools. This is achieved by simply executing the annotation tool of interest on a set of contact maps sampled by HiCSampler and capturing the variance of the predictions. Since predictions based on each sample are independent, all of them can be conducted in parallel, adjusting the number of samples to achieve the desired level of distribution accuracy.

To the best of our knowledge, HiCSampler is the first method developed for the uncertainty measurement of Hi-C data. However, there remains room for improvement. Despite having optimized the acceptance rate by introducing the adaptive proposal and auxiliary variables, the Metropolis–Hastings algorithm still has a low acceptance rate. We believe that revising the model in order to replace the Metropolis–Hastings sampler by a Gibbs sampler can significantly speed up the sampling procedure. The size of the matrices analyzed and the complexity of the MCMC inference also make HiCSampler relatively slow. Variational inference (Blei et al., 2017) and GPU-based implementations could provide significant speedups. Second, HiCSampler utilizes an empirical Bayes method to infer the prior distribution of the interaction frequency. Although this works well in practice, it is at risk of overfitting in theory. Fully Bayesian approaches could provide a more robust inference. Although Hi-C datasets with multiple replicates are becoming available and are being analyzed using different tools in recent years (Yang et al., 2017; Stansfield et al., 2019), HiCSampler currently only infers posterior distributions of interaction frequencies based on a single Hi-C contact map. In the future, it will be of interest to extend HiCSampler to model variability measured across multiple replicates of Hi-C data. In addition, we can expand HiCSampler to incorporate epigenetic and functional genomics features. For example, since CTCF-binding sites play an important role in chromatin loop formation, we can update the prior by modifying the local potential to encourage contact pairs enriched by CTCF-binding sites to have a higher interaction frequency.

In conclusion, HiCSampler enables a detailed analysis of uncertainty in contact frequency estimation and in downstream annotation and 3D structure prediction tasks. Capturing uncertainty in 3D genomics is particularly important, considering the relatively high degree of stochastic noise caused by the relatively low sequencing compared to the size of the contact matrices being estimated. We expect that this will enable the robust and statistically sound analysis of HiC data.

## Data Availability

Publicly available datasets were analyzed in this study. These data can be found here: GSE63525, https://www.ncbi.nlm.nih.gov/geo/query/acc.cgi?acc=GSE63525.
